# Editorial: Tools for assessing family relationships

**DOI:** 10.3389/fpsyg.2025.1599368

**Published:** 2025-05-08

**Authors:** Marialuisa Gennari, Stephen E. Finn, Alessandra Maria Roberta Santona, Giancarlo Tamanza

**Affiliations:** ^1^Department of Psychology, Catholic University of the Sacred Heart, Milan, Italy; ^2^Center for Therapeutic Assessment, Austin, TX, United States; ^3^Department of Psychology, University of Milan-Bicocca, Milan, Italy

**Keywords:** family relations assessment, clinical assessment, clinical relational interviews, family questionnaires, observational assessments, interactional assessments

Family relationships are one of the most interesting and significant areas of study in clinical psychology. The family, as the first and most important context for human development, is composed of affective and interactive dynamics that profoundly shape individuals, influencing their socio-emotional wellbeing, mental health, and relationship skills. These influences persist across the entire lifespan.

The inherent complexity of family relationships emerges from the recognition that each family system is a dynamic entity, composed of unique individuals with distinct perceptions, needs, and personal stories. Their reciprocal interactions give rise to a relational framework that is systemic and constantly evolving. Therefore, understanding family dynamics is a highly complex undertaking that requires a rigorous and diversified methodological approach. A multidimensional perspective is indispensable, highlighting the internal representations and lived experiences of different family members, as well as the quality of the family relationships and interactions, through a variety of investigative instruments and constructs.

Our main goal in curating this Research Topic was to present some of the most recent tools for studying the multifaceted nature of family functioning from a relational perspective. A secondary objective was to elicit contributions from researchers around the world. Indeed, understanding family relationships cannot be disentangled from the cultural context in which they develop. Caregiving practices, social expectations, and the meanings attributed to affective bonds vary significantly across cultures, making it essential to adapt and validate assessment instruments within specific contexts (see contributions by Rinaldi et al.; Aschieri, Cera et al.; Velotti et al.; Shek et al.).

Another important element we held in mind was that multiple perspectives must be considered within a family system. To navigate this complexity, research on family relationships has traditionally used both quantitative and qualitative approaches and frequently incorporates mixed-method designs that combine these ways of understanding family systems. This methodological integration allows for the exploration of family dynamics from complementary angles, leading to a deeper and more nuanced picture of how families work.

Among the widely used quantitative methodologies, self-report questionnaires stand out. These instruments, completed individually by family members (grandparents, parents, or children), facilitate the collection of standardized information regarding their internal working models, attitudes, and experiences related to family relationships. An example is the Italian validation of the CPRS-I by Rinaldi et al., which assesses parental perceptions of closeness, conflict, and dependence in the parent-child relationship. Similarly, the Parent Experience of Assessment Scale (QUEVA-G) by Aschieri, Brasili et al. focuses on parental satisfaction with the psychological assessments of their children and their relationship with the assessors. The Chinese Family Assessment Instrument (C-FAI) by Shek et al. is a self-report measure designed to evaluate adolescents' perceptions of their family's functioning. The Parental Stress Scale, studied in its Russian version by Bochaver et al., specifically examines parental stress.

Clinical interviews are an important tool for in-depth exploration of family narratives, the meanings attributed to relationships, and the history of the family system. Their flexibility and capacity to delve into specific themes enable researchers and clinicians to grasp the nature of family experiences, construct a shared narrative of relational dynamics, and uncover partially unconscious meanings and experiences. The Clinical Generational Interview (CGI), described by Tamanza and Gennari, is a structured interview that assesses family relationships based on the construct of family generativity. Through a series of questions and pictorial stimuli, the CGI reconstructs family history, couple dynamics, and parental expectations via a dialogue with the parental couple, analyzing intergenerational bonds and transmission processes. The Current Relationship Interview (CRI), whose Italian validation was examined by Velotti et al., focuses instead on romantic relationships, drawing from attachment theory to evaluate individuals' internal working models in their current intimate relationships. The CRI may be used with married or unmarried couples. The Adult Attachment Projective Picture System (AAP) by George and Wargo Aikins is a performance-based test that is administered individually; it has proven to be an efficient, rigorously validated tool for assessing internal representations of attachment. The AAP is valuable for therapists who seek to understand dysfunctional family processes and formulate therapeutic goals.

Along with self-report instruments and qualitative interviews, observational methods play a crucial role in studying family interactions. These approaches allow for the direct analysis of behavioral and relational dynamics in real time, offering insights into communication patterns, interactive sequences, and the quality of exchanges among family members. Direct observation, supported by structured coding systems, overcomes the limitations of subjective reports by capturing relational dynamics that might not be consciously reported by family members. In this regard, the Triadic Interactional Analytical Procedure (TIAP), described by Cigala et al., exemplifies an observational methodology that examines micro-interactions among family members in different configurations, revealing fundamental aspects of family functioning. This tool assesses a family system's ability to cope with developmental tasks, communicate effectively, establish clear and flexible rules, and provide support to its members. Similarly, the Marschak Interaction Method of Psychometrics (MIM-P) and the Assessment of Parent–Child Interaction (APCI), whose psychometric properties were studied by Jacobsen et al., are observational tools designed to evaluate caregiver-child relationships through structured tasks and the analysis of non-verbal and affective interactions.

Interactive graphic tools provide an additional view of family relationships, by facilitating the expression of complex and unconscious relational dynamics, especially in contexts where verbal communication may be limited or challenging. An example is the Family Life Space (FLS), described by Gennari et al.. This task actively engages all family members in the joint creation of a drawing representing their family system. The analysis of the drawing, together with the observation of interactions during the creative process, provides valuable insights into relational quality, power dynamics, feelings of belonging, and potential areas of conflict or emotional distance within the family.

In closing, we believe that the diverse articles assembled for this Research Topic demonstrate that the intricate and fascinating mosaic of family relationships can only be fully understood through a multimethod, multidimensional perspective that combines both individual and systemic elements. Such an approach illuminates both the richness and complexity of family systems and is helpful to researchers and clinicians alike.

